# Nonculturable Escherichia coli O157 in horticultural compost: a public health concern

**DOI:** 10.1099/acmi.0.001090.v4

**Published:** 2025-10-16

**Authors:** Callum Highmore, C. William Keevil

**Affiliations:** 1 School of Biological Sciences, University of Southampton, Southampton, UK; 2 National Biofilms Innovation Centre (NBIC) and Institute for Life Sciences, University of Southampton, Southampton, SO17 1BJ, UK

**Keywords:** *Escherichia coli* O157, microbial detection, viable but nonculturable (VBNC)

## Abstract

Fresh produce-associated outbreaks of the foodborne pathogen *Escherichia coli* O157 are responsible for a number of disease cases, hospitalizations and deaths. In many cases, the source of contamination can be linked to the growing media of the food, although pathogen detection is problematic in these complex soil ecosystems. In this study, direct quantitative real-time PCR without pre-enrichment was used to detect 310 copies of the Tir gene, using a primer sequence specific to *E. coli* O157, in horticultural compost purchased from a commercial supplier. The pathogen could not be cultured on selective media but was visualized using peptide nucleic acid fluorescence *in situ* hybridization and cell elongation viability assay, confirming the viability. Enumeration of elongated *E. coli* O157 determined that there were 205 live cells per gram of compost. The nonculturability and confirmation of viability of the pathogen indicates its viable but nonculturable (VBNC) status. The detection of VBNC foodborne pathogens in environmental samples challenges current understanding of the nature of foodborne pathogen contamination.

## Data Summary

All relevant data used in this study are presented within the manuscript, and no data upload to an external repository is required to reproduce this work.

## Introduction

Fresh produce is a prominent vehicle by which foodborne pathogens infect humans, causing more disease cases than any other food commodity and accounting for 38% of hospitalizations [1]. Enterohaemorrhagic *Escherichia coli* (EHEC), particularly serovar O157:H7, is a prominent foodborne pathogen and was the causative agent for multistate disease outbreaks associated with lettuce in 2019, hospitalizing 46% of patients [2]. It also caused three further outbreaks in the USA in 2021, associated with fresh produce or unknown food sources [3].

Ingestion of *E. coli* O157 can result in infection causing abdominal cramps and bloody diarrhoea, with some patients developing haemolytic–uraemic syndrome due to the presence of Shiga-like toxins [4]. The ability of EHEC O157 to cause disease is facilitated by its attachment to the intestinal epithelium mediated by the products of a major pathogenicity island found in the genome called the locus of enterocyte effacement (LEE). LEE encodes proteins that form a specialized type III secretion system that injects EHEC proteins such as translocated intimin receptor (Tir) into the host cell as well as the other injected proteins. Tir subsequently migrates to the enterocyte surface and facilitates binding of EHEC O157 via the intimin protein on the pathogen’s surface, also expressed from the LEE pathogenicity island. This initiates remodelling of the enterocyte cytoskeleton and rearrangement of the actin filaments underneath the bacterial adhesion site, resulting in a deformation of microvilli into a dysfunctional pedestal-like structure designated the site of attachment and effacement. The *tir* gene varies in length between different pathogenic *E. coli* serovars from 538 (*E. coli* O26:H-) to 558 aa (*E. coli* O157:H7), making it a useful marker for identification studies [5].

The pathogen can enter a viable but nonculturable (VBNC) state, a survival state in which physiological processes are altered so that the bacterium can endure a range of physical and chemical stressors at the expense of its ability to replicate under laboratory conditions [6,7]. In the agricultural environment, VBNC induction of *E. coli* O157 has been observed when inoculated onto the lettuce phylloplane at low temperatures [8], excluding it from commonly used culture-dependent detection methods [9,10]. However, Truchado *et al.* found that VBNC *E. coli* O157 inoculated onto lettuce leaves could not be resuscitated after 15 days incubation [11]. There is evidence that *E. coli* O157 can retain its pathogenicity while in the VBNC state; Liu *et al.* identified the expression of the Shiga-like toxin gene 19 months after VBNC induction [12].

Soil is often considered to be one of the sources of *E. coli* O157 contamination [13,14]. The pathogen is able to contaminate vegetables through its contact with the soil by being splashed onto the phylloplane by rain or irrigation [15,17], by uptake by the rhizosphere permitting transmission to the leaf tissues [18] and even by insect vectors [19]. As a pathogen of the gastrointestinal tract, *E. coli* O157 can be disseminated across agricultural land through defecation by roaming animals and deliberate application of manure as fertilizer, further spread across agricultural land by heavy rainfall. Cattle manure is commonly used to this end and cattle comprise the primary reservoir for the pathogen, where some ‘super-shedding’ individuals have been reported to shed >10^4^ c.f.u. *E. coli* O157:H7/g faeces [20]. While the relationship between super-shedding cattle and contamination of farmland is poorly understood, their presence has been linked to the dissemination of *E. coli* O157:H7 between farms and to rates of human infection [21].

VBNC *E. coli* O157 could persist in the soil long after it loses culturability while maintaining disease potential. The persistence of * E. coli* O157:H7 under starvation conditions has been previously studied, where the viability of one tested strain was unaffected after 84 days of incubation in PBS [22]. *E. coli* O157 incubated in soil-extracted soluble organic matter for 24 days demonstrated an altered gene expression profile, suggesting that it is capable of maintaining stable populations in soil for extended periods of time [23]. VBNC induction could occur through prolonged exposure to low temperatures in soil, which has been explored in previous studies [24,25], though often in conjunction with nutrient deprivation. The existence of VBNC *E. coli* O157 has been indicated in Japanese river water [26]. Detection of a range of VBNC pathogens in a range of environmental samples has previously been established; however, this has primarily been achieved by inoculating bacteria into samples and assessing culturability rather than direct analyses manipulating environmental isolates [27,29].

This study aims to detect VBNC *E. coli* O157 in horticultural compost using *tir* gene detection with quantitative real-time PCR (qPCR) and validated by microscopy, to demonstrate the ubiquity of the pathogen in the environment and the complex relationship between the VBNC state and the threat it poses to food safety.

## Methods

### Bacteria and growth conditions

Bacteria used for qPCR were non-pathogenic *E. coli* O157:H7 NCTC 12900, which lacks Shiga-like toxin genes. Before inoculation, they were grown to a concentration of 5×10^7^ c.f.u. ml^−1^ in brain heart infusion broth (Oxoid, UK) for 18 h at 37 °C. Samples were diluted in PBS (Oxoid).

Cell elongation was carried out using a modification of the method by Juhna *et al.* by adding 1 ml sample to 5 ml R2 broth (0.1% w/v peptone, 0.05% w/v yeast extract, 0.05% w/v glucose, 0.05% w/v starch, 0.03% w/v potassium dihydrogen phosphate, 0.03% w/v sodium pyruvate and 0.0024% w/v magnesium sulphate), 4 ml ddH_2_O and 100 µl pipemidic acid at a concentration of 1 mg ml^−1^. The sample is incubated for 22 h at 22 °C in darkness and concentrated by centrifuging 10 ml sample for 15 min at 4,000 ***g*** using a Heraeus Megafuge 1.0 and resuspending in 1 ml PBS prior to PNA-FISH [30].

### Compost sample preparation

Analysed material was peat-based compost from a commercial supplier (Wickes, UK). Compost samples were either left ‘pristine’, i.e. unsterilized and uninoculated with bacteria, or they were sterilized. Compost was sterilized by spreading in a thin layer and autoclaving using a Priorclave benchtop autoclave at 123 °C for 30 min. Positive control samples were inoculated with either 10^6^, 10^4^ or 10^3^ c.f.u. * E. coli* O157 per gram of compost sample prior to pulsification. Samples of 25 g were added to BagFilter P stomacher bags (Interscience, France), 225 ml ddH_2_O was added and the sample was pulsified using a Pulsifier™ (Microgen Bioproducts Ltd., UK) for 30 s. Compost samples were then concentrated by filtration as previously described [31] for downstream qPCR experiments. Compost samples were not concentrated ahead of PNA-FISH experiments.

### PNA-FISH procedure

Compost samples of 50 µl were spread across Cyclopore 0.2 µm pore membranes (Whatman, UK), dried and fixed with 90% (v/v) ethanol. Cells were hybridized using 50 µl hybridization buffer (10% w/v dextran sulphate, 10 mM NaCl, 30% v/v formamide, 0.1% w/v sodium pyrophosphate, 0.2% w/v polyvinylpyrrolidone, 0.1% v/v Triton X-100, 0.2% w/v Ficoll, 5 mM disodium EDTA and 50 mM TrisHCl) with 200 nM PNA probe. Membranes were incubated in darkness for 90 min at 59 °C and then washed by soaking in wash buffer (5 mM NaCl, 1% v/v Triton X-100) in darkness for 30 min at 59 °C. Probe used was EcoPNA1169, targeting *E. coli* O157 23S ribosome with the sequence 5′-CAA CAC ACA GTG TC-3′ [32], with fluorescent marker Alexa Fluor 546, with absorption maximum 556 nm and emission maximum 573 nm. The probe did not detect *Enterobacter cloacae* ATCC 13047, *Acinetobacter baumannii* W1 and *Streptococcus pneumoniae* D39. Slides were viewed at 1,000 times magnification using oil immersion and episcopic differential interference contrast/epifluorescence microscopy.

### Enumeration and image analysis

Enumeration of elongated cells in pristine compost was carried out by counting the total number across 100 fields of view and multiplying the average number of cells by the average area of a compost suspension droplet (7.01 mm^2^) to find the number of cells per compost suspension sample. This was used to calculate the number of cells per gram of compost sample. Calculation of droplet area was carried out using ImageJ 1.46r.

### DNA extraction and qPCR assay

DNA extraction of samples used Powersoil DNA Isolation kit (Mo Bio, USA) according to the manufacturer’s instructions. qPCR was carried out according to Genesig *E. coli* O157:H7 kit instructions (Primer Design, UK, product discontinued), targeting the *tir* gene specific to *E. coli* O157 labelled with an FAM dye. The qPCR method consisted of a 10-min warming stage at 95 °C and then 50 cycles of 10 s at 95 °C followed by 60 s at 60 °C. A Bio-Rad iQ5 cycler was used and copy numbers were calculated using a standard curve containing known *tir* gene copy numbers. The target gene was quantified by measuring the sample Ct, the number of amplification cycles required for the fluorescence generated by the target gene amplification to exceed the background fluorescence. Statistical analyses of qPCR data used one-way ANOVA with Tukey’s multiple comparison test, and an unpaired two-tailed t-test carried out using GraphPad Prism 7.

### Attempted isolation of *E. coli* O157 from compost

Samples of pristine compost weighing 5 g were incubated in 50 ml modified tryptone soya broth (mTSB) (Oxoid) supplemented with a selective supplement containing vancomycin, cefixime and cefsulodin (Sigma-Aldrich, USA) for 24 h at 37 °C. Samples were concentrated by filtration onto a mixed cellulose-ester membrane with a pore size of 0.22 µm (Millipore) and were resuspended in 8 ml PBS. The sample was divided into eight aliquots of 1 ml each for immunomagnetic separation using Dynabeads anti-*E. coli* O157 (Thermo Fisher, USA) according to the manufacturer’s instructions. The resulting suspension was plated onto CHROMagar O157 in its entirety (CHROMagar, France) and incubated for 24 h at 37 °C. Mauve colonies were considered positive results.

## Results

### qPCR detection of *E. coli* O157 genes in compost

**Fig. 1.**
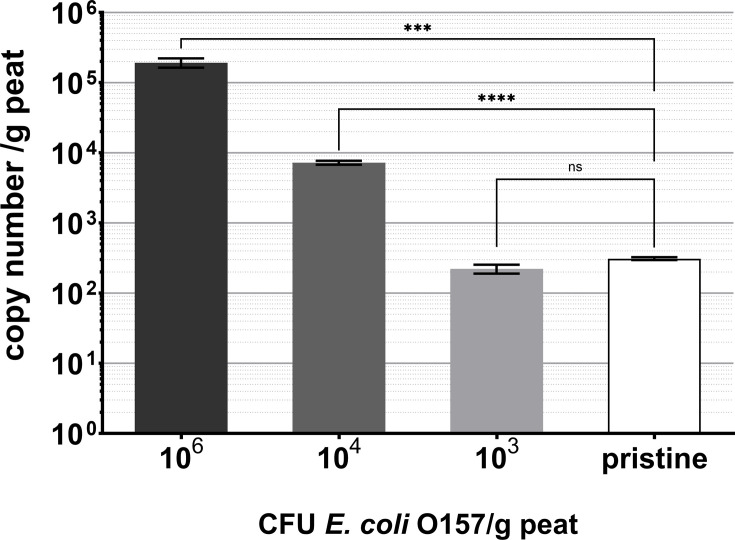
*tir* gene copy numbers of *E. coli* O157 inoculated into peat-based compost and detected in pristine peat-based compost. Samples inoculated with known concentrations of bacteria were sterilized prior to inoculation, and pristine samples were not sterilized, with no bacterial inoculation. Statistical difference refers to t-test comparisons, with *** showing *P*=0.0007 and **** showing *P*<0.0001. ns shows non-significance. Error bars indicate sem of 4–6 technical replicates.

In sterilized compost samples inoculated with 10^6^ c.f.u. *E. coli* NCTC 12900 per gram, 1.9×10^5^ *tir* gene copies were detected per gram of compost, 7,261 were detected in compost samples inoculated with 10^5^ c.f.u. g^−1^ and 222 were detected in samples inoculated with 1,000 c.f.u. g^−1^ (Fig. 1). A one-way ANOVA determined that there was a statistical difference between the detected copy number (*P*<0.0001). In pristine compost samples, 310 *tir* gene copies were detected per gram of compost. A t-test determined that the number of *tir* genes in compost samples inoculated with 1,000 c.f.u. g^−1^ was not statistically different compared to uninoculated, unsterilized pristine compost samples (*P*=0.0578, *n*=5), indicating that a population of *E. coli* O157 was present in the pristine compost at a similar concentration to compost samples with 1,000 c.f.u. g^−1^ *E. coli* 12900 inoculated into it (Fig. 1). A background of 31 *tir* gene copies detected in sterilized, uninoculated compost samples was subtracted from the total *tir* gene copy number of all samples.

### Detection of *E. coli* O157 using PNA-FISH

**Fig. 2.**
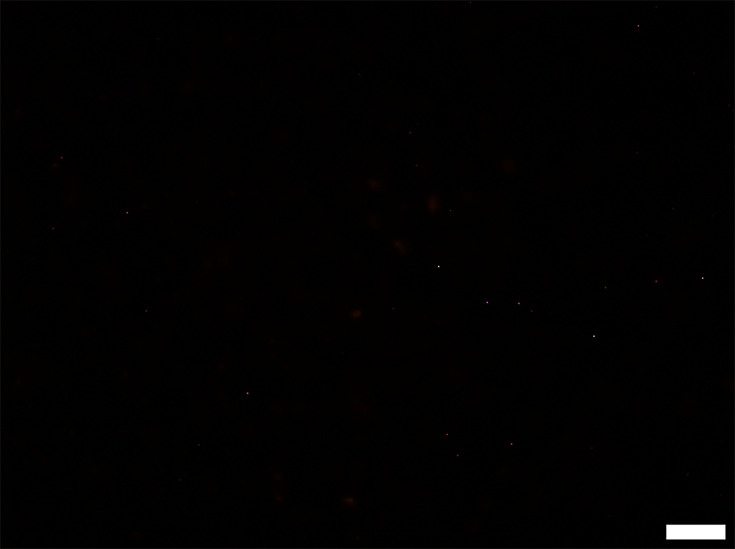
Representative epifluorescence micrograph of sterilized compost. Scale bar represents 10 µm.

**Fig. 3.**
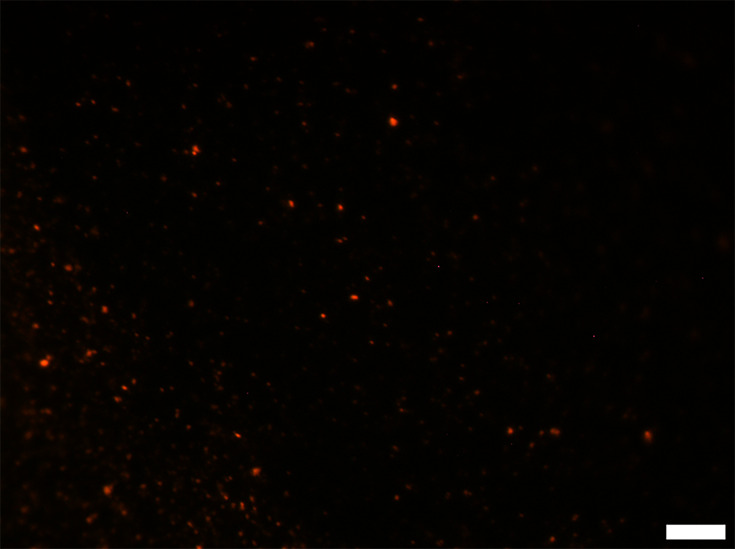
Representative epifluorescence micrograph of sterilized compost inoculated with 10^6^ c.f.u. *E. coli* O157/g. Scale bar represents 10 µm.

Sterilized peat-based compost samples were assessed as a negative control, using the *E. coli* O157-specific PNA probe (Fig. 2). The samples contained no visible living cells, with dim auto-fluorescence of some soil particles within the compost. When the sterilized compost was spiked with 1.8×10^6^ *E. coli* O157/g as a positive control, brightly fluorescing cells were visible throughout the sample, indicative of a high rRNA content (Fig. 3). Additionally, some cells appeared to fluoresce less brightly than others.

**Fig. 4.**
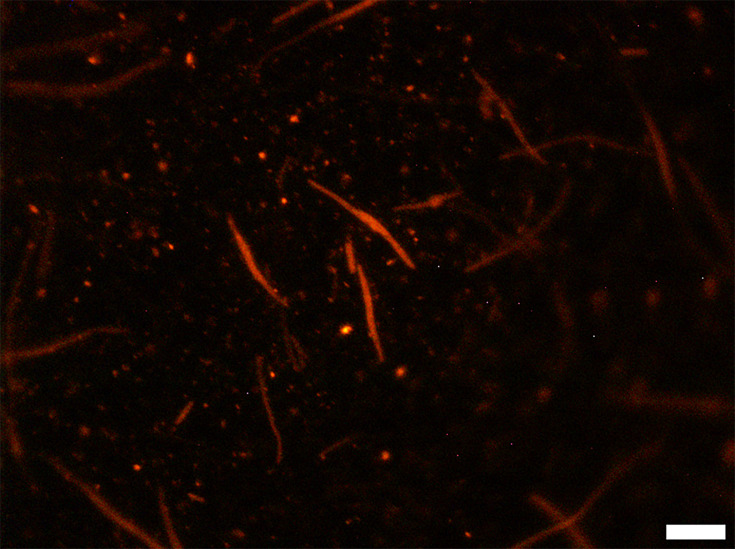
Representative epifluorescence micrograph of sterilized compost inoculated with 10^6^ c.f.u. *E. coli* O157/g and incubated to induce cell elongation. Scale bar represents 10 µm.

**Fig. 5.**
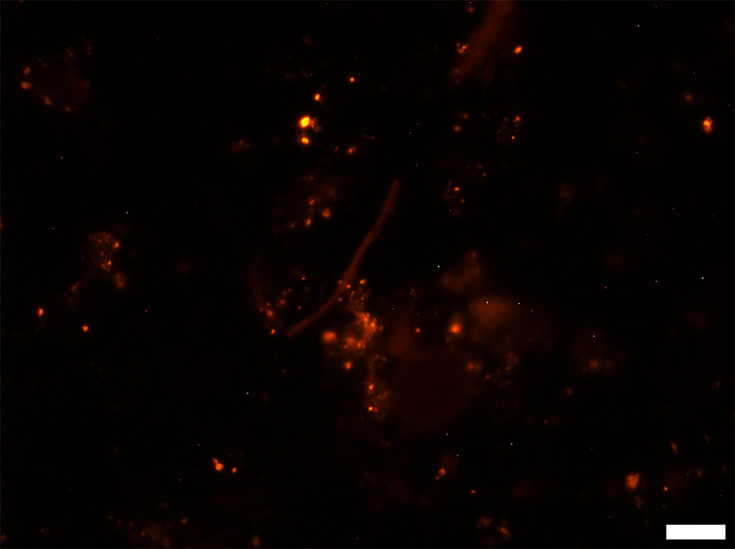
Representative epifluorescence micrograph of pristine compost incubated to induce cell elongation. Scale bar represents 10 µm.

Inoculated compost samples that had undergone cell elongation treatment displayed highly elongated cells, in some cases to lengths greater than 30 µm. Despite this, some cells did not elongate, with varying degrees of fluorescence (Fig. 4). Exposing pristine compost to the cell elongation treatment permitted the visualization of fluorescing, elongated cells (Fig. 5). Six elongated cells were counted across 100 fields of view in pristine compost samples, corresponding to 205 cells per gram of compost sample.

Attempts to isolate and culture the *E. coli* O157 cells were unsuccessful, where samples were incubated with mTSB (Oxoid) at 37 °C for 24 h. The cultures underwent immunomagnetic separation before plating on CHROMagar O157. This suggests that the fluorescent * E. coli* O157 detected by the PNA probe was VBNC, as they responded to cell elongation treatment but did not grow on culture media. Conducting thorough VBNC resuscitation procedures was not possible in this study due to the polymicrobial nature of the compost samples and the inability to isolate the pathogen using immunomagnetic separation.

## Discussion

While many studies observe that VBNC pathogens are capable of persisting in the environment, this research confirms the presence of VBNC *E. coli* O157 in horticultural compost. Confirmation of VBNC *E. coli* O157 in pristine peat-based compost was determined by PNA-FISH and cell elongation, qPCR and an absence of colonies on culture media (Figs1  5). Our previous work has detected the *tir* gene at lower concentrations in six other soil types by the same qPCR methodology, but these soils were not investigated further for the source of the detected gene [30]. Other studies have determined that the VBNC state can be induced in *E. coli* O157 [7,8] and that it can continue the expression of virulence factors such as Shiga-like toxin gene *stx1* while in this state [12]. *E. coli* O157, along with other foodborne pathogens including *Listeria monocytogenes*, has been resuscitated from the VBNC state and even implicated in outbreaks of foodborne disease [33,34]. These factors present a risk to public health and pose a challenge to the agricultural industry, although the resuscitation and isolation of the pathogen was not possible in this study.

The VBNC state is currently defined by an inability for bacterial cells to grow on routine, nutrient-rich media, irrespective of inducing stressor or molecular marker. The absence of common molecular markers is an enduring barrier to the recognition of the VBNC state, caused in part by the range of inducing stressors, both physical and chemical, and by the range of bacterial species that can enter the state [35]. Recently explored VBNC detection methods including the use of propidium monoazide in combination with qPCR (PMA-qPCR) [11,36, 37] demonstrate an effective and improved technique for quantification of viable pathogen contamination in food samples, but the labour required makes the adoption of this technique into the food sector unlikely. Hyperspectral microscopy mediated by artificial intelligence has been used to identify VBNC *E. coli* cells in conjunction with viability staining [38], which may prove effective at scale in future but is not currently used in industry.

The discrepancy between the cells inoculated into the compost and the *tir* gene copy number retrieved from the qPCR assay (Fig. 1) is caused by the loss of bacterial sample to the filter membrane during vacuum filtration [31] and may be contributed to by DNA adherence to residual compost particles and the presence of humic acids [39,40]. However, known concentrations of bacteria inoculated into sterilized peat-based compost act as a standard curve, estimating total *tir* gene copy number in pristine compost at 1,113 *tir* gene copies per gram. Similarly, using PNA-FISH, it was calculated that pristine compost contained 205 VBNC *E. coli* O157 cells per gram; correcting for sample loss to the filter membrane, the total number of *E. coli* O157 cells in pristine compost is estimated at 978 cells per gram. The discrepancy between the qPCR and PNA-FISH data can be attributed to the low number of *E. coli* O157 in the sample and the assumption that there is an even distribution of cells across the compost sample when an uneven distribution is far more likely. Alternatively, fewer cells could have been quantified by PNA-FISH as the bacteria may have been present at different depths or obscured by organic matter from the compost. The detected *E. coli* O157 cells could have originated from animal manure or wastewater contamination present at the peat bog source of the compost. The estimated concentration of cells is high, and survival of *E. coli* O157 in fertilizer-amended soils including peat has previously diminished to the 100 c.f.u. g^−1^ limit of detection in 15 days [41]; however, this study used enumeration by culture and did not account for the potential of VBNC persistence. Additionally, a fraction of the *tir* gene copies could come from dead cells remaining in the compost.

Previous studies have determined that bacterial foodborne pathogens have the capacity to continue expression of virulence factors while in the VBNC state and following resuscitation inside a host [12,42,44]. Although rare, there have been *E. coli* O157 disease cases thought to have been caused by contaminated compost in the garden; one outbreak led to four cases of the disease following consumption of vegetables grown in compost containing the pathogen [45]. Outbreaks with no identifiable source could suggest VBNC contribution to foodborne disease [46,48]. The current understanding of the VBNC state is that it represents a continuum that encompasses a range of nonculturable states [49,50]. That principle holds in the present study, where it was found that *E. coli* O157 readily elongated under the stringent conditions of R2 broth (Fig. 5) but would not grow on culture media.

In the future, this work could be conducted at a larger scale to investigate the distribution of *E. coli* O157 contamination in a range of soil types from different geographic origins. More comprehensive work to resuscitate and isolate pathogens from soil, along with other viability assays such as PMA-qPCR, could better determine the extent of the ‘nonculturable’ pathogen reservoir in soil. Although this study determined the identification of *E. coli* O157 using qPCR together with PNA-FISH, the complementarity of the *E. coli* O157 *tir* gene to other pathogenic *E. coli* such as *E. coli* O55 and *E. coli* O145 presents the potential for there to be other EHEC strains present in the tested compost samples. Detection of Shiga-like toxin genes would also help to confirm the pathogenicity of VBNC EHEC detected in these samples. Future work should include more thorough genomic or metagenomic sequencing approaches of environmental samples to complement microbiological techniques that confirm the VBNC status of pathogens present. Further research is also needed to examine the relative culturability of dormant pathogens in the environment and to assess the infectivity of those pathogens present in the farm-to-fork chain, where microbial safety is dependent on pathogen detection by culture.

## Abbreviations

EHEC: enterohaemorrhagic *Escherichia coli*
LEE: locus of enterocyte effacement
mTSB: modified tryptone soya broth
Tir: translocated intimin receptor
VBNC: viable but nonculturable
